# Impact of Alligator Weed (*Alternanthera philoxeroides*) Invasion on Floral Composition and Soil Microbiota

**DOI:** 10.1155/sci5/7359416

**Published:** 2025-12-18

**Authors:** Shristi Khanal, Hari Sharan Adhikari, Mukesh Kumar Chettri

**Affiliations:** ^1^ Department of Botany, Amrit Campus (Tribhuvan University), Kathmandu, 44600, Nepal; ^2^ Department of Botany, Bhaktapur Multiple Campus (Tribhuvan University), Bhaktapur, 44800, Nepal

**Keywords:** allelopathic effect, plant invasion, plant–soil feedback, soil microbes, soil microbial activity

## Abstract

Plant invasion modifies the aboveground and belowground biota directly or indirectly via allelopathic effect. This study aimed to ascertain if the invasive species *Alternanthera philoxeroides* impacts plant diversity, plant composition, and soil microbes or not. The soil microbial activity (CO_2_ release) and soil pH were also recorded. The plant communities invaded and uninvaded with *Alternanthera philoxeroides* were examined using the quadrat method. Soil samples were collected from 0 to 10 cm depth, and the culture method was used for soil microbial analysis. The plant species richness and soil fungi were found to be reduced at *A. philoxeroides* invaded plots than at uninvaded plots. The IVI of all common species such as *Cynodon dactylon*, *Bidens pilosa,* and *Trifolium repens* was highly suppressed in the invaded zone. The parameters like plant diversity indices, colony count of soil microbes, soil microbial activity (CO_2_ release), and soil pH were found to be reduced at invaded sites than at uninvaded sites. The results indicated that the invasive *A. philoxeroides* modifies the plant community composition, and the underlying mechanism for the change is possibly by altering the soil microbiota, microbial respiration, and soil pH with their successful invasion.

## 1. Introduction

Over the past several decades, the global spread of naturalized and invasive plants has surged dramatically, reshaping ecosystems across continents. Invasion ecology highlights that invasive alien species often succeed by harnessing preadaptive traits and undergoing rapid adaptive evolution once introduced [1]. As a consequence, many such species substantially disrupt native ecological processes in meadows, steppes, wetlands, and forests, leading to the formation of monodominant communities that diminish biodiversity and challenge native wildlife [2]. Yet, the specific mechanisms driving these invasions and the long‐term ecological consequences of species naturalization remain complex and difficult to predict [3].

One notable example is *Alternanthera philoxeroides* (Martius) Griseb., commonly known as alligator weed, a perennial herb of the Amaranthaceae family native to the Paraná River region of South America [4]. This species primarily reproduces vegetatively through fragments of stem, rhizome, or root tubules, which are easily dispersed by water. It thrives in shallow waters and marshes along roadsides, ditches, drainage areas, and pond and lake edges, as well as in agricultural fields. Alligator weed degrades wetlands, reduces crop production, and replaces valuable forage species in pastures [5, 6]. In north New Zealand lakes, *A. philoxeroides* has been shown to alter macrophyte decomposition rates [7], while in agricultural lands, it causes significant yield reductions in various crops, including rice (45%), wheat (36%), sweet potato (63%), lettuce (47%), and corn (19%) [8, 9].

As an amphibious plant, *A. philoxeroides* outcompetes native species by occupying both aquatic and terrestrial niches, forming dense mats that reduce light penetration into the water and suppress native plant growth [10, 11]. This species decomposes rapidly than native plants, altering the nutrient cycling and ecosystem processes of invaded communities and often facilitating the colonization of additional invasive species [7]. Moreover, the species produces aqueous allelochemicals that inhibit the growth of surrounding vegetation, particularly affecting aquatic ecotypes [12].

The invasion history of alligator weed underscores its global impact. It was first recorded in the USA in 1897 [13] and began to emerge as a serious weed by the 1960s [14]. Introduced in China during the 1930s as a forage crop, it now occurs widely throughout the country [15]. In Nepal, *A. philoxeroides* was first reported as an invasive alien plant in 1994 and has since spread across altitudes ranging from 80 to 1350 m, where it is recognized as a problematic weed in both agricultural and wetland ecosystems [16–21]. Interestingly, species diversity within *A. philoxeroides* invaded communities is higher in terrestrial ecosystems compared to wetland areas [22].

Invasion ecology emphasizes that plant invasions are not only a matter of species displacement but also of altered ecosystem functioning. Soil microbes, which play an essential role in nutrient cycling and plant community dynamics [23, 24], are particularly sensitive to invasive plants. Their activities and populations are regulated by various factors, including soil properties, vegetation cover, and climatic conditions [25, 26]. Invasive plant species such as alligator weed are known to alter soil moisture, temperature [27], pH, microbial activity, and nutrient availability [28, 29], thereby potentially disrupting native plant–microbe interactions and altering ecosystem resilience.

Despite the rapid expansion of *A. philoxeroides* in agricultural and wasteland ecosystems of Nepal [30], its ecological impacts remain insufficiently understood. It is still unclear whether alligator weed is a primary driver of biodiversity decline and altering soil microbial communities. Therefore, this study investigates the effect of *A. philoxeroides* on both the plant and soil microbial communities, aiming to improve predictions of agricultural land function under alligator weed invasion and contribute to more effective management strategies.

## 2. Materials and Methods

### 2.1. Study Site

**Figure 1:**
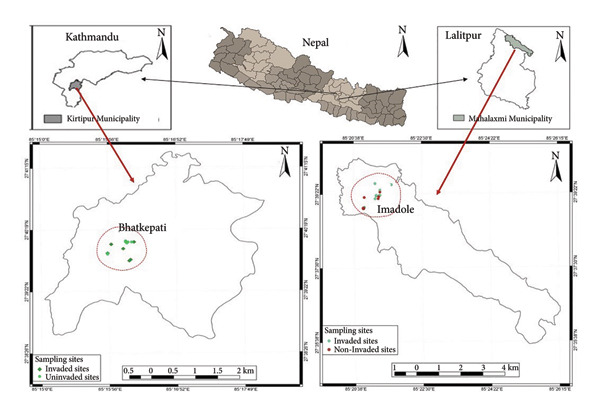
Map showing study area (Bhatkepati and Imadole) inside Kirtipur Municipality of Kathmandu District and Mahalaxmi Municipality of Lalitpur District.

Kathmandu Valley is in the central region of the country, lying between the Mahabharat and high Himalayas. Two places, Imadole and Bhatkepati in Kathmandu Valley, were selected for the present study (Figure 1). Bhatkepati lies at an elevation ranging from 1300 to 1390 m above sea level in Kirtipur Municipality of Kathmandu District and experiences a subtropical climate, i.e., gently warm in summer and cold in winter. The latitude and longitude of Bhatkepati are 27°40′ N and 85°16′ E, respectively. Imadole is a part of Mahalaxmi Municipality in Bagmati Province of Central Nepal, located in Lalitpur District, having a climate similar to Bhatkepati, and its coordinates are 27°6 N and 85°35 E. The meteorological data recorded at the nearest weather station (Khumaltar) for 5 years (2015–2018) showed maximum and minimum monthly average temperatures in June (28.96°C) and January (2.78°C), respectively. The highest precipitation was recorded in July (294.26 mm) and the lowest in November (less than 1 mm).

### 2.2. Sampling Design and Soil Sample Collection

A random sampling design was employed in the wasteland of Bhatkepati (Kathmandu) and Imadole (Lalitpur). The study sites included both *A. philoxeroides* invaded quadrats and uninvaded quadrats. A total of 40 plots (1 × 1 m^2^) were laid at each study site including invaded and uninvaded quadrats. The sampling quadrats with more than 75% coverage of *A*. *philoxeroides* were considered as invaded, whereas sampling quadrats with less than 10% coverage of other invasive species were considered as uninvaded.

Vegetation data were collected from each quadrat where the identification was confirmed by comparing specimens with those housed at National Herbarium and Plant Laboratories (KATH), Godavari, Lalitpur, as well as with records from the World Flora Online (http://www.worldfloraonline.org/).

Soil samples were collected from a depth of 0–10 cm (top soil) at five points within each quadrat (four corners and one from the center). Composite representative samples were prepared from each sampling quadrat. These samples were sealed air tight in sterilized zip‐lock polythene bags, immediately transported to laboratory, and stored at −10°C in refrigerator for further analysis.

### 2.3. Soil Microbe Culture, Isolation, and Identification

The method given by Waksman [31] and Aneja [32] was followed for the soil dilution plate method. From the dilutions, 1 mL inoculum was poured onto potato dextrose agar (PDA) medium‐plated Petri plates and incubated at 25 ± 2°C for 7 days–10 days. The number of colonies was counted and fungi were isolated. The isolated colonies were identified based on standard literature [33, 34]. For bacteria, nutrient agar (NA) medium was used and incubated at 37°C for 24 h, 48 h, and 72 h. The colony forming unit (CFU/g) was calculated from those colonies by using the following formula.

(1) CFU/g=n×d,

where n = number of colonies and d = dilution factor = 1/dilution.

### 2.4. Soil pH and Soil Microbial Activity

Soil pH of soil samples was measured by using a digital glass electrode. The carbon dioxide release from soil microbial activity was carried out following the method described by Zobel et al. [35].

### 2.5. Data Analysis

The Importance Value Index (IVI) is a comprehensive quantitative tool that assesses the ecological significance of species in a community. To compute IVI, the following formula [36] was used for each species.

(2) IVI=relative frequency+relative coverage+relative density,

where relative frequency, relative coverage, and relative density refer to the percentages of one species frequency, coverage, and density over the sum of all species coverage, frequency, and total density within a plot, respectively.

Shannon–Wiener diversity index, Simpson’s diversity index, and Sorenson’s similarity index (SSI) were calculated for the evaluation of species diversity, species richness, and evenness of ground vegetation in invaded and uninvaded sites using the standard equation given by Magurran [37].

(3) Shannon index H=−∑pilnpi pi=niN,

where pi = proportion of individuals found in species I, n_i_ is the number of individuals in species i, and N is the total number of individuals in the community.

(4) Simpson’s index D=1−∑n−1NN−1,

where n = number of individual species and N = total number of individuals in the community.

SSI [38] was calculated using the following formula:

(5) Sorenson’s similarity index SSI=2Ca+b+2c,

where a = number of species in community A; b = number of species in community B; and C = number of species common to both the communities.

The microbial counts (CFU), pH, and CO_2_ data obtained were statistically analyzed using an independent *t*‐test. All the data analysis and graph preparation were done in MS Excel 2016. Regression analysis was used to evaluate the relationship between different vegetation and soil parameters.

## 3. Results

### 3.1. Effect on Floral Composition

**Figure 2:**
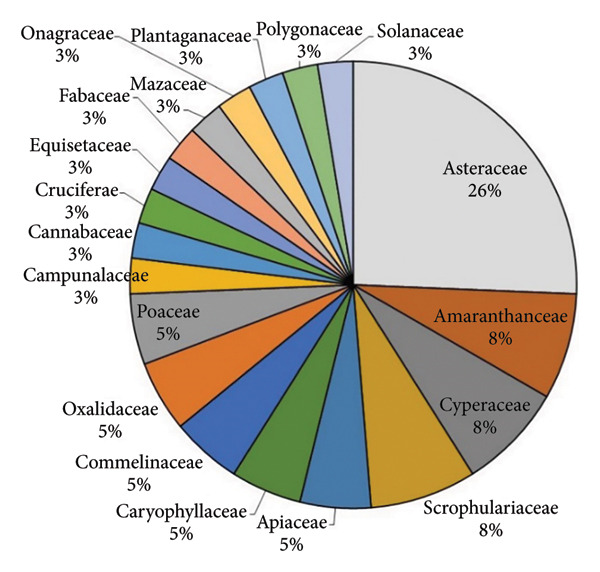
Percentage of different families present in study sites.

Altogether 39 species of plants from 19 families were recorded in study sites. Among them, Asteraceae comprised the highest number of species (10 spp.) and Amaranthaceae (3 spp.), Cyperaceae (3 spp.), and Scrophulariaceae (3 spp.) occupied second rank. Asteraceae showed the highest percentage followed by Amaranthaceae (8%) and Cyperaceae (8%). Solanaceae showed the lowest percentage (1 sp.) comprising the lowest number of species followed by Polygonaceae (1 sp.) (Figure 2). In Bhatkepati, 35 species were listed, out of which 34 species were recorded at uninvaded quadrats and only eight species at *A*. *philoxeroides* invaded quadrats. Similarly, 32 species were recorded at uninvaded quadrats of Imadole and only 10 species at *A*. *philoxeroides* invaded quadrats. *Trifolium repens* was the dominant plant species in Bhatkepati uninvaded sites, whereas *Lobelia chinensis* was in uninvaded sites of Imadole. *A*. *philoxeroides* was a highly dominant species in invaded sites in both study sites. A total of six other invasive plant species (i.e., *Ageratum conyzoides, Ageratum haustonianum, Bidens pilosa, Galinsoga quadriradiata, Parthenium hysterophorus,* and *Oxalis latifolia*) were also reported.

The IVI of *C. rotundus* and *T. repens* was the highest in Bhatkepati, whereas it was the lowest in Imadole. Similarly, *A. paniculata*, *L. chinensis,* and *C. dactylon* showed the highest IVI in Imadole (Figure 3). The IVI of *A. philoxeroides* was the highest at both Bhatkepati and Imadole. The IVI of *Ageratum conyzoides*, *Bidens pilosa*, *Trifolium repens*, and *Cynodon dactylon* was recorded comparatively much less than that of *Alternanthera philoxeroides* in invaded plots (Figure 3).

Figure 3Importance Value Index (IVI) of five common species in *Alternanthera philoxeroides* uninvaded (Un) and invaded (In) sites of Bhatkepati and Imadole.

**Figure:**
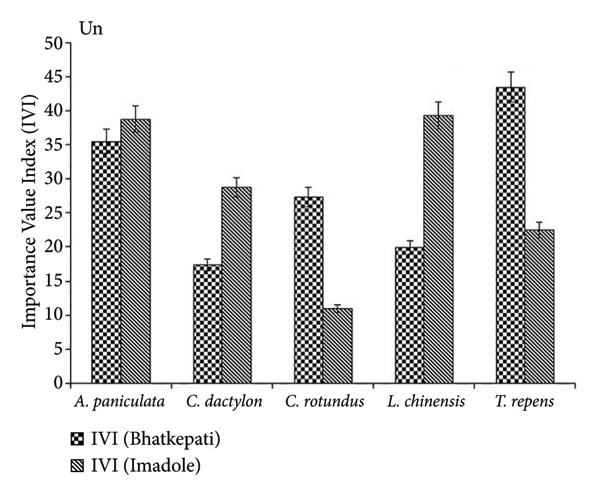
(a)

**Figure:**
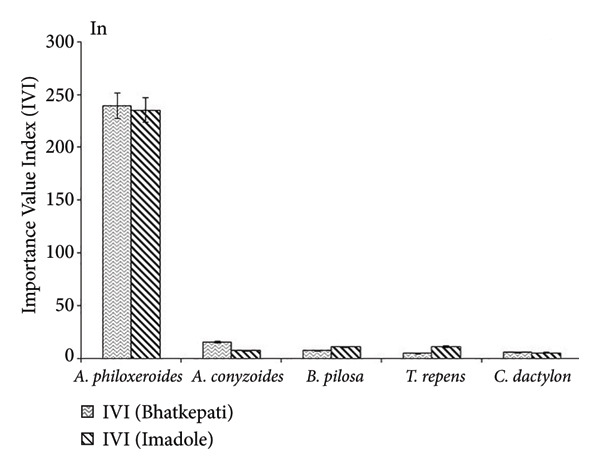
(b)

The coverage of *A. philoxeroides* and species richness of plant species was compared with Bhatkepati and Imadole (Table 1). The coverage of *A*. *philoxeroides* was very high in both invaded sites. Few numbers of other species were present in invaded sites. Due to the highest percentage of cover of invasive plants of *A*. *philoxeroides*, species richness was decreased in both Bhatkepati and Imadole.

**Table 1:** Cover and number of noninvasive species in *A. philoxeroides* invaded quadrats.

Study sites	Coverage of *A. philoxeroides* (mean ± SD)	Number of noninvasive species (mean ± SD)
Bhatkepati	92.05 ± 7.97	2.25 ± 1.25
Imadole	93.20 ± 3.26	2.10 ± 1.22

The Shannon–Wiener index and Simpson’s index of Bhatkepati and Imadole were different. The highest values of both indices were recorded in the uninvaded sites of Bhatkepati, while the lowest values were observed in the invaded sites (Table 2). A similar pattern was found in Imadole (Table 2).

**Table 2:** Species diversity indices.

S.N.	Diversity indices	Uninvaded		Invaded	
		Bhatkepati	Imadole	Bhatkepati	Imadole
1.	Shannon–Wiener diversity index	2.71	2.54	0.32	0.24
2.	Simpson’s diversity index	0.91	0.88	0.12	0.07

The index of similarity was highest (0.86) between the uninvaded sites of Bhatkepati and Imadole. This was followed by the invaded sites (0.77) of Bhatkepati and Imadole. The higher the index value, more similar the stands to each other. The similarity index between invaded and uninvaded sites of Bhatkepati was less (0.33) which was followed by an index (0.35) between invaded sites of Bhatkepati and uninvaded sites of Imadole (Table 3).

**Table 3:** Sorenson’s similarity index of total plant species present in uninvaded and invaded quadrats.

Study sites		Bhatkepati		Imadole	
		Invaded	Uninvaded	Invaded	Uninvaded
Bhatkepati	Invaded	—			
	Uninvaded	0.33	—		

Imadole	Invaded	0.77	0.40	—	
	Uninvaded	0.35	0.86	0.42	—

The regression analysis (Figure 4) showed a somewhat positive relationship (*R* ^2^ = 0.2096) between the coverage of other invasive species and species richness of noninvasive species per quadrat in *A. philoxeroides* uninvaded areas of Bhatkepati. Meanwhile, such a relationship was also weakly positive (*R* ^2^ = 0.0387) in Imadole. Likewise, coverage of other invasive species has a weakly positive (*R* ^2^ = 0.0702) relationship with the species richness of other invasive species in Bhatkepati. Meanwhile, such a relationship was somewhat positive (*R* ^2^ = 0.1746) in Imadole.

Figure 4Relationship of other invasive species (not *Alternanthera philoxeroides*) coverage with noninvasive species diversity (a and c) and other invasive species (b and d) at uninvaded quadrats in Kirtipur (a and b) and Imadole (c and d) study sites.

**Figure:**
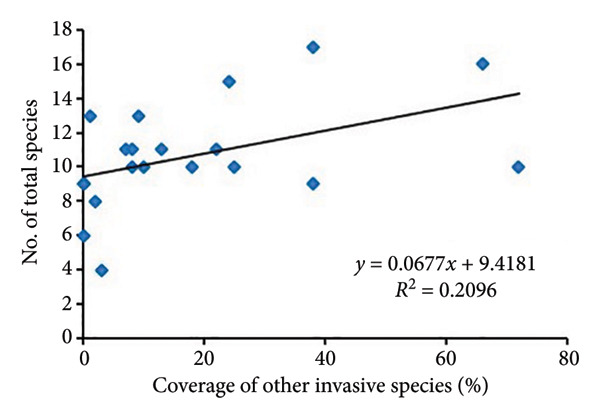
(a)

**Figure:**
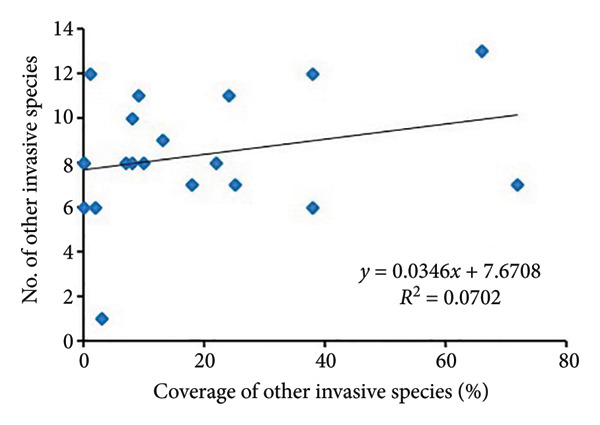
(b)

**Figure:**
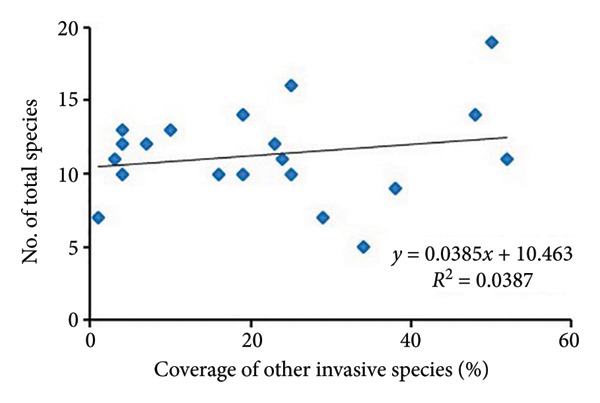
(c)

**Figure:**
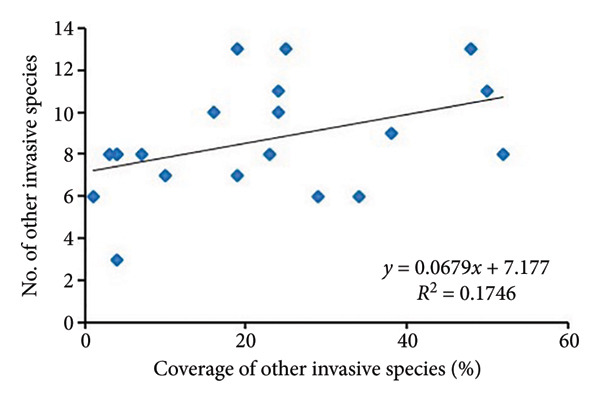
(d)

### 3.2. Effect on Soil Microbiota

The colony number (per gram) of fungi showed insignificant difference (*p* > 0.05) in uninvaded and invaded soil types of Bhatkepati and Imadole. Meanwhile, the number of bacterial colonies was significantly (*p* > 0.05) different in Bhatkepati but it was statistically insignificant (*p* > 0.05) in Imadole (Table 4). Independent sample *t*‐test showed that there was an insignificant difference in colonies of fungi in uninvaded soil and invaded soil. Meanwhile, some bacterial colonies were found to be significantly different (Table 4).

**Table 4:** Number of microbial colonies in the soil of uninvaded and invaded quadrats.

Study area	Type of soil	Fungal colonies/g			Bacterial colonies/g		
		(Mean ± SD)	*t* value^a^	*p* value	(Mean ± SD)	*t* value^a^	*p* value
Bhatkepati	Uninvaded	89.56 ± 31.37	1.28	0.218	90.44 ± 20.05	4.119	0.001
	Invaded	73.80 ± 21.93			55.30 ± 17.15		

Imadole	Uninvaded	85 ± 26.38	1.08	0.296	60.71 ± 14.14	0.502	0.623
	Invaded	69.45 ± 31.64			54.45 ± 30.75		

^a^ *t* value obtained from independent *t*‐test (*n* = 39).

The colony forming unit (CFU/g) was observed higher in uninvaded soil of both Bhatkepati (3.8 × 10^4^) and Imadole (36 × 10^4^). Meanwhile, it was less in the invaded soil of both Bhatkepati (27 × 10^4^) and Imadole (28 × 10^4^) (Figure 5(a)). A total of 26 soil fungal species belonging to 17 genera (12 families) were recorded from uninvaded and invaded quadrats at Bhatkepati and Imadole. The fungal diversity was higher in *A*. *philoxeroides* uninvaded soil than in *A*. *philoxeroides* invaded soil in both research sites. The species richness of soil fungi was higher in uninvaded soil (22 and 18 each) than in invaded soil (10 and 14 each) in both research sites (i.e., Bhatkepati and Imadole) (Figure 5(b)).

Figure 5Comparison of CFU/g (a) and number of soil fungi (b) present in *Alternanthera philoxeroides* invaded and uninvaded plots of each study site.

**Figure:**
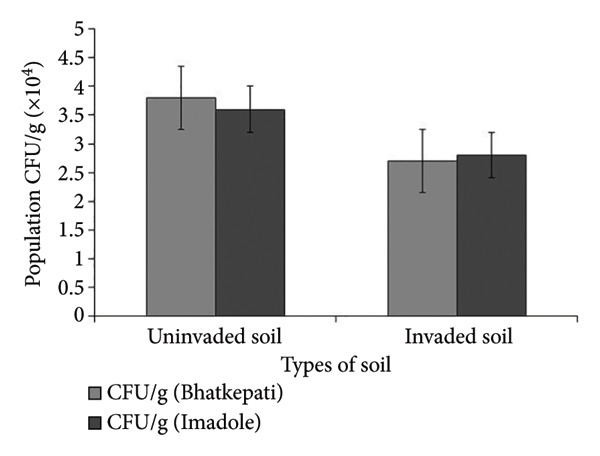
(a)

**Figure:**
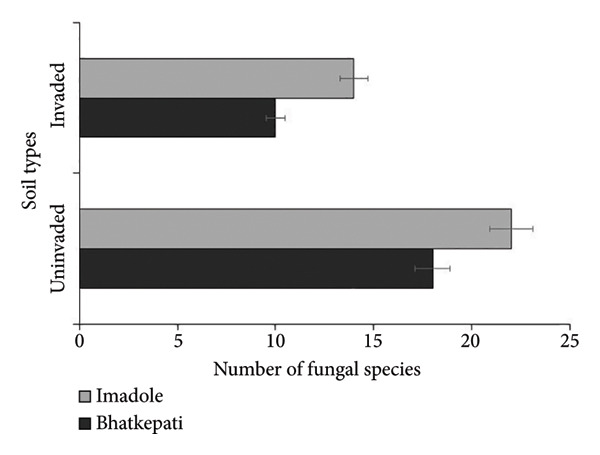
(b)

In *A*. *philoxeroides* uninvaded soil, the most frequent fungal species were *Mucor circinelloides* (100%), *Penicillium chrysogenum* (100%), *Fusarium solani* (88.89%), and *Aspergillus niger* (44.44%) in Bhatkepati, whereas it was *Penicillium chrysogenum* (100%), *Fusarium* sp. (85.72%), *Aspergillus niger*, *Cladosporium* sp., *Trichoderma harzianum* (71.43%, each), and *Fusarium oxysporum* (57.14%) in Imadole (Figures 6(a) and 6(b)). But in case of *A*. *philoxeroides* invaded soil, the most frequent fungal species were *Mucor circinelloides* (88.89%), *Fusarium solani* (77.79%), *Aspergillus niger* (66.67%)*, Trichoderma harzianum* (55.56%), and *Aspergillus flavus* (44.44%) in Bhatkepati, whereas it was *Aspergillus niger*, *Penicillium chrysogenum* (100% each), *Fusarium oxysporum* (72.72%), *Fusarium solani* (36.36%), and *Trichoderma harzianum* (27.27%) in Imadole (Figures 6(b) and 6(d)). Meanwhile, the fungal species such as *Alternaria alternata*, *Aspergillus flavus*, *Aspergillus niger*, *Cladosporium* sp., *Fusarium oxysporum*, *Fusarium solani*, *Mucor circinelloides*, *Paecilomyces* sp., *Penicillium* spp., *Rhizopus stolonifer*, *Trichoderma harzianum*, and *Trichoderma viride* were reported common in both uninvaded and invaded sites (Figure 6).

Figure 6Occurrence of fungal species in uninvaded soil of Bhatkepati (a), invaded soil of Bhatkepati (b), uninvaded soil of Imadole (c), and invaded soil of Imadole (d).

**Figure:**
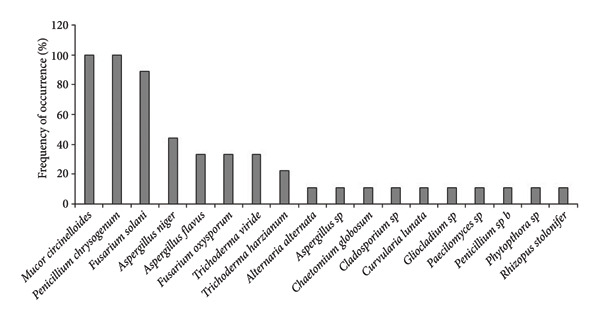
(a)

**Figure:**
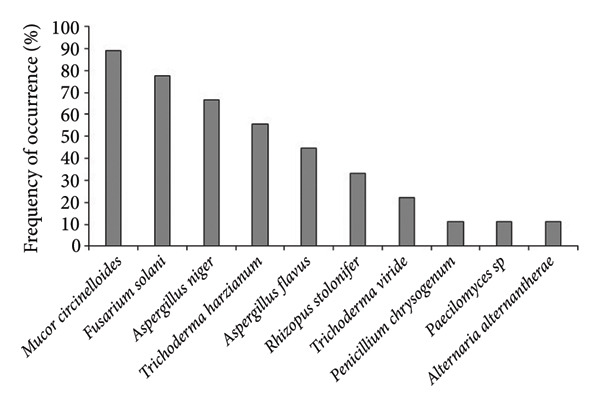
(b)

**Figure:**
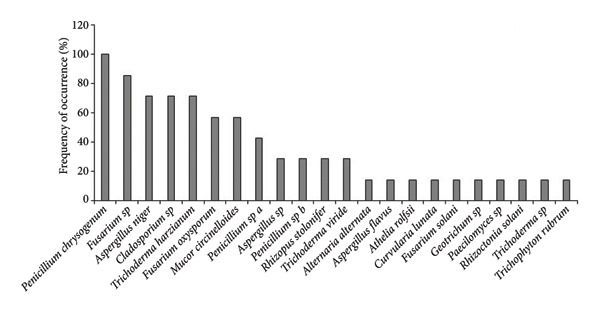
(c)

**Figure:**
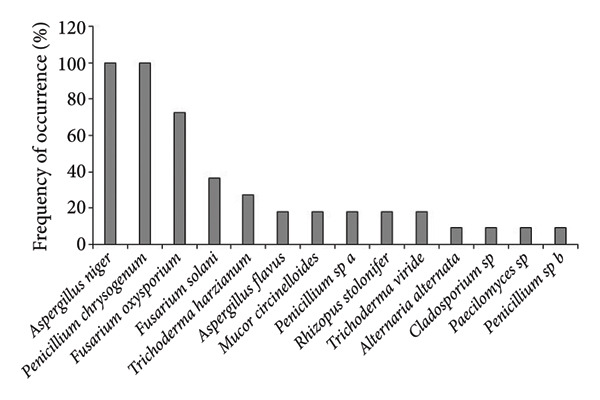
(d)

### 3.3. Effect of *Alternanthera philoxeroides* Invasion on Soil pH and Soil Microbial Activity

There was a significant difference (*p*  <  0.05) in the pH of uninvaded and invaded soil types of Bhatkepati and Imadole (Table 5). Independent sample *t*‐test showed that there was a significant difference in mean pH value with that of soil type in uninvaded soil and invaded soil. There was a high significant difference (*p*  <  0.05) in CO_2_ released from uninvaded and invaded soil types of Bhatkepati and Imadole (Table 6).

**Table 5:** pH value of soil of uninvaded and invaded quadrats.

Study area	Type of soil	pH value (mean ± SD)	*t* value^a^	*p* value
Bhatkepati	Uninvaded	6.82 ± 0.497	7.34	0.0001
	Invaded	5.55 ± 0.212		

Imadole	Uninvaded	6.50 ± 0.319	6.54	0.0001
	Invaded	5.68 ± 0.211		

^a^t value obtained from independent *t*‐test (*n* = 39).

**Table 6:** CO_2_ release (mg/100 g of soil) from soil microbial activity.

Study area	Type of soil	CO_2_ release (mean ± SD)	*t* value^a^	*p* value
Bhatkepati	Uninvaded	30.37 ± 1.79	10.64	0.0001
	Invaded	20.23 ± 2.29		

Imadole	Uninvaded	27.47 ± 2.059	9.02	0.0001
	Invaded	17.66 ± 2.35		

^a^t value obtained from independent *t*‐test (*n* = 39).

## 4. Discussion

### 4.1. Impact on Floral Diversity and Composition

Based on the above results, the invasion of *A*. *philoxeroides* has shown a significant impact on the floral diversity and composition, as relatively very low species richness was recorded in invaded sites in comparison to that of uninvaded sites at both study areas. Such influence of invasive plants on species richness might be due to various means such as sequestration of resources, direct competition, and modification of ecological processes [12, 39, 40]. Invasive plants adopt many strategies to be successful in a community which is amplified by climate change [41]. *A. philoxeroides* can quantify range expansion and boost population density. As it spreads by vegetative fragmentation and waterborne dispersal of propagules [6, 42], it displaces other plant species and disrupts ecological processes [43, 44]. The coverage of other invasive plants present in uninvaded sites showed less impact resulting in high species richness of associated species in *A*. *philoxeroides* uninvaded sites. Due to the high coverage of *A*. *philoxeroides* in invaded sites, associated species were highly affected. *A. philoxeroides* decreases the stability of the plant community and displaces native species [7, 16, 45]. The phenotypic plasticity of *A. philoxeroides* to cope with adverse ecological stresses and other resources, especially in the early stages of their life cycle, has been speculated for its successful invasion [46, 47]. The low diversity indices in invaded plots at both research sites might also possibly be due to the high phenotypic plasticity of *A. philoxeroides* on the one hand and the effect of allelochemicals like flavones, terpenes, sterols, and ethyl propionate on the other [12, 48]. In this study, the consistent presence of multiple invasive plants like *Ageratum conyzoides*, *Bidens pilosa,* and *Galinsoga quadriradiata* across *A. philoxeroides* invaded plots indicates the facilitative interactions between *Alternanthera philoxeroides* and other invasive species [11].

### 4.2. Impact on Microbial Activity and Composition

The soil health appears to be significantly influenced by soil microorganisms, an integral component of the soil ecosystem [49]. Invasive species may affect the soil’s health by releasing allelochemicals that are harmful to some native plants that depend on microbial communities for growth [23]. Higher species richness of soil fungi in the uninvaded soil than in the invaded soil in the current study indicated that *A. philoxeroides* invasion altered the soil microbiota. These results supported the hypothesis regarding the alteration of the soil biota by plant invasion, provided by various researchers [15, 50, 51]. The results also explained the previously ambiguous relationships between soil microbes and plant invasion [52]. Some previous studies [44, 53, 54] showed that *A. philoxeroides* enhanced the number of mycorrhizal soil fungi, and possibly this enhanced mycorrhizal colonization produced a positive feedback loop to improve the invasiveness of *A. philoxeroides*. By comparing the findings of the current experiment with those of previous investigations, it is shown that *A. philoxeroides* can replace numerous groups of soil fungi that are frequently present in soil that are not overrun by weeds. Occurrence frequency in this study showed a similar tendency of less species richness of pathogenic and saprophytic fungi in the invaded soil which is supported by Ge et al. [55], while it was contradictory with the verdict of some other previous studies [44, 53, 54]. Plants can affect the rhizosphere microbiota by rhizodeposition [56]. The presence of allelochemicals such as 4‐hydroxy‐3‐methoxybenzoic acid, *m*‐coumaric acid, tannins, flavonoids, saponins, ethyl propionate, and ethyl acetate in *A. philoxeroides* [12, 44, 57] could account for changes in the number of soil fungal and bacterial colonies, the species composition of soil fungi, and the loss in species richness.

Soil pH plays a vital role in bacterial and fungal growth and soil enzyme activity [58]. The soil pH, organic content, and moisture are the main factors that affect the population and diversity of fungi [59, 60]. In this present study, the number of colonies of bacteria is reduced in invaded sites which might be due to the low pH value seen in invaded sites. Similar results were also observed by Rousk et al. [61], who reported that the composition of the bacterial communities is affected by pH showing a direct influence on bacterial community composition. CO_2_ released decreased significantly at invaded sites than at uninvaded sites indicating less microbial activity at invaded sites. This study indicates that *A. philoxeroides* modifies the microbial activity in the soil which is possibly due to the exudation of allelochemicals named ethyl propionate [57]. In this way, *A. philoxeroides* not only threatens plant biodiversity in agroecosystems [30, 62] but also is problematic in different Ramsar sites [18, 20, 21] and forests [19] as well. So, its management is crucial for the conservation of soil health of agroecosystem and other ecosystem functioning [62].

This study offers initial insights into soil microbial dynamics under *A. philoxeroides* invasion. However, reliance on culture‐dependent methods limits the detection of total microbial diversity. Although resource constraints precluded molecular analysis, future studies should incorporate high‐throughput sequencing to provide a more comprehensive understanding.

## 5. Conclusion

From the vegetation analysis and biodiversity indices, it can be concluded that *A. philoxeroides* reduces the plant species diversity in invaded areas. The invasion of *A. philoxeroides* reduced species richness and diversity in invaded communities. Due to the high coverage of *A. philoxeroides*, the number of species was reduced severely in the invaded sites of Bhatkepati and Imadole. From the CO_2_ release due to microbial activity in soil and microbial colony count experiments, it can be concluded that *Alternanthera* modifies the environment that favors its growth and this may help in the process of invasion. Though *A. philoxeroides* is not listed as the worst invasive alien plant species, it is spreading rapidly in terrestrial habitats (agricultural fields, abandoned land, and barren land) day by day. It may become noxious weeds in the future if appropriate measures for the management of *A. philoxeroides* are not taken in time and might cause threats to biodiversity and economic losses in Nepal.

## Conflicts of Interest

The authors declare no conflicts of interest.

## Funding

The authors received no specific funding for this work.

## Data Availability

The data that support the findings of this study are openly available in ResearchGate at https://doi.org/10.13140/RG.2.2.27586.70083.
